# Chronic Dietary Exposure of Roosters to a Glyphosate-Based Herbicide Increases Seminal Plasma Glyphosate and AMPA Concentrations, Alters Sperm Parameters, and Induces Metabolic Disorders in the Progeny

**DOI:** 10.3390/toxics9120318

**Published:** 2021-11-24

**Authors:** Loïse Serra, Anthony Estienne, Guillaume Bourdon, Christelle Ramé, Claire Chevaleyre, Philippe Didier, Marine Chahnamian, Souleiman El Balkhi, Pascal Froment, Joëlle Dupont

**Affiliations:** 1 Centre National de la Recherche Scientifique, Institut Français du Cheval et de l’Equitation, Institut National de Recherche pour l’Agriculture, l’Alimentation et l’Environnement, Université de Tours, PRC, F-37380 Nouzilly, France; loise.serra@inrae.fr (L.S.); Anthony.estienne@inrae.fr (A.E.); guillaume.bourdon@inrae.fr (G.B.); Christelle.rame@inrae.fr (C.R.); Claire.chevalere@inrae.fr (C.C.); pascal.froment@inrae.fr (P.F.); 2 Institut National de Recherche pour l’Agriculture, l’Alimentation et l’Environnement—Unité Expérimentale du Pôle d’Expérimentation Avicole de Tours UEPEAT 1295, F-37380 Nouzilly, France; Philippe.dider@inrae.fr (P.D.); Marine.Chahnamia@inrae.fr (M.C.); 3 Service de Pharmacologie, Toxicologie et Pharmacovigilance, CHU, F-87042 Limoges, France; Souleiman.bakic@inrae.fr

**Keywords:** glyphosate, Roundup, fertility, endocrine disruptors, male chicken, progeny

## Abstract

The effects of chronic dietary Roundup (RU) exposure on rooster sperm parameters, fertility, and offspring are unknown. We investigated the effects of chronic RU dietary exposure (46.8 mg kg^−1^ day^−1^ glyphosate) for 5 weeks in 32-week-old roosters (*n* = 5 RU-exposed and *n* = 5 control (CT)). Although the concentrations of glyphosate and its main metabolite AMPA (aminomethylphosphonic acid) increased in blood plasma and seminal fluid during exposure, no significant differences in testis weight and sperm concentrations were observed between RU and CT roosters. However, sperm motility was significantly reduced, associated with decreased calcium and ATP concentrations in RU spermatozoa. Plasma testosterone and oestradiol concentrations increased in RU roosters. These negative effects ceased 14 days after RU removal from the diet. Epigenetic analysis showed a global DNA hypomethylation in RU roosters. After artificial insemination of hens (*n* = 40) with sperm from CT or RU roosters, eggs were collected and artificially incubated. Embryo viability did not differ, but chicks from RU roosters (*n* = 118) had a higher food consumption, body weight and subcutaneous adipose tissue content. Chronic dietary RU exposure in roosters reduces sperm motility and increases plasma testosterone levels, growth performance, and fattening in offspring.

## 1. Introduction

Glyphosate (G), N-(phosphonomethyl) glycine, is largely used as the active ingredient of commercial herbicides such as Roundup (RU). It has a broad spectrum, acting on around 100 different weed species and around 60 perennial weed species [1]. In animals as in plants, G is transformed into CO_2_ and amino-methyl-phosphonic acid (AMPA) by glyphosate oxidoreductase [2]. For a long time, G has not been described as dangerous for mammals and humans because the shikimate pathway is not found in vertebrates [2]. However, multiple emerging studies show that G and glyphosate-based-herbicides (GBHs) have harmful effects on vertebrates at different levels, such as the central nervous system, the blood, the metabolism of the liver, the kidney or the reproductive system [1,3,4,5,6]. In mammals, for example, male mice exposure to GBHs induces a delay of the testis descent [7], increased sperm abnormalities and a decrease in sperm motility [8]. Moreover, GBHs are suspected to act as endocrine disruptors. Indeed, GBHs impair normal hormonal functions. Steroidogenesis in mammals is disturbed, with reduced levels of steroidogenic acute regulatory (StAR) protein amount and testosterone (T) in male rodents [9,10,11,12] and perturbations of oestrogen (E2) and progesterone (P) secretion in female rodents [13,14] and in female piglets [15]. In addition, AMPA can also inhibit cell growth and decrease the viability of ovarian SKOV-3 and OVCAR-3 cell lines [1]. Sopko et al. (2021) [16] have recently tested the effects of chronic exposure to G (0.7 and 7 mg/L) for 100 days on spermatogenesis in rats and showed that G was able to interact with the cycle-specific eukaryotic translation elongation factor 1 subunit alpha 1 (eEF1α1), leading to protein synthesis delay and spermatogenesis and cell growth suppression [16]. However, our understanding of the underlying molecular mechanisms involved in cellular responses to G or GBHs needs to be improved.

Many avian species are highly impacted by human activities. For example, 70% of the cultures species are threatened by exposure to pesticides [17]. This exposure could be due to accidental exposure because of industrial uses or to the deliberate use of pesticides. In a previous study, exposure to pesticides such as di(n-butyl) phthalate (DBP) via the food for 30 days in adult Japanese quails (*Coturnix japonica*) perturbed enzymes involved in steroidogenesis, including StAR protein, cytochrome P450 side-chain cleavage (P450scc), P450c17 (CYP17), CYP19 (P450 aromatase) and 3β-hydroxysteroid dehydrogenase (3 β-HSD) [18]. Long-term exposure to GBHs, with a concentration of G below that causing observable adverse effects (NOAEL; 100 mg/kg/ body weight/day; [19]), in Japanese quail showed that G and AMPA were found within the liver and reduced the T levels at puberty in males [20]. Moreover, GBHs may have epigenetic effects, impacting the offspring when the parents are exposed [21]. The latter study highlighted that G residues were found in eggs and that GBHs caused both lipid damages within the brains of embryos and poor embryonic development. In adult drakes (*Anas platyrhynchos*), exposure to GBHs for 15 days caused a reduced level of plasma T and E2 as well as a reduction in the epithelium of the seminiferous tubules (ST) and interstitial tissue, leading to an altered epididymis morphology [22]. Finally, studies focusing on the in ovo exposure of chicken embryos found that GBHs promoted embryonic mortality [23], reduced the percentage of hatching [24], disrupted cytochrome P450 enzymes in the liver and small intestine [25] and increased reactive oxygen species (ROS) production [24,25]. Only a few studies have been performed on the effects of GBHs in avian species. In addition, according to our knowledge, no studies were conducted on the effects of chronic dietary GBH exposure or the fertility of the male chicken via offspring analysis.

Based on the literature, we tested the hypothesis that dietary RU exposure can alter not only sperm motility and testis steroidogenesis but also fertility and the growth development of the offspring. Thus, the objectives of this study were to investigate the impacts of chronic GBH exposure and, more precisely, of RU via food exposure on sperm parameters, plasma testosterone and oestradiol levels, fertility and growth and fattening of the progeny.

## 2. Materials and Methods

### 2.1. Ethical Issues

All experimental procedures were performed in accordance with the French National Guidelines for the care and use of animals for research purposes (certificate of authorisation to experiment on living animals APAFIS number 21549-2019071809504554v3, Approval Date: 6 November 2021, Ministry of Agriculture and Fish Products, and a notice of ethics committee of Val de Loire N◦19).

### 2.2. Animals

**Figure 1:**
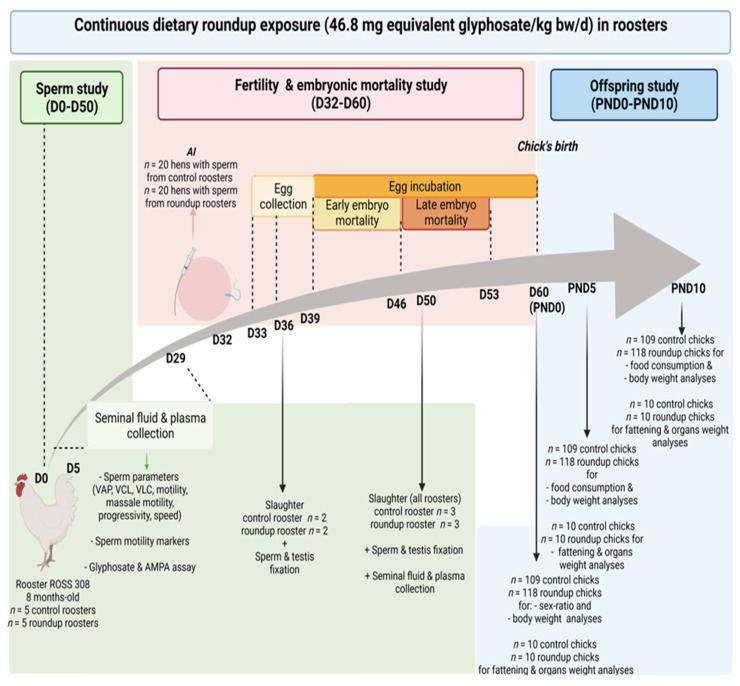
Experimental design.

All 308 ROSS animals (10 roosters and 40 hens) were obtained at 1 day of age from a local hatchery (Boye Accouvage La Villonniere 79310 La Boissière en Gatine, France) and reared at “Pôle Expérimental Avicole de Tours” (INRAE, Nouzilly, France) according to the traditional breeding conditions. In our experiment, 10 roosters 32 weeks old and 40 hens 32 weeks old were used. After artificial insemination of the hens, we also studied 227 chicks. All animals were killed by electrical stunning and bled out, as recommended by the ethical committee. The design of the experiment is summarised in Figure 1.

The timeline is represented in days (D). (AI: artificial insemination; VAP: average path velocity; VSL: straight-line velocity; VCL: curvilinear velocity. PND: postnatal day. bw/d: body weight per day.) Ten 8-month-old roosters ROSS 308 were included in the study. Five RU roosters were exposed for 36 days to RU via the food (46.8 mg/kg bw/d), and five CT roosters were fed with a regular diet without RU. During this period (D0 to D36), from D5 to D25, blood and the sperm samples of all roosters were collected to analyse sperm parameters and to quantify glyphosate and its metabolite AMPA within the seminal fluid and the blood plasma. At D32, 20 hens were artificially inseminated with sperm from control roosters and 20 hens with sperm from RU roosters. The next day and for 6 days, eggs were collected. At D36, exposure to RU was stopped, and two CT roosters and two RU roosters were slaughtered to examine testis morphology. At D50, all other roosters were slaughtered to perform the same analyses. During this time, eggs fertilised with the sperm of CT or RU roosters were incubated from D39 for 21 days. At D46, early embryo mortality was assessed by candling, followed by assessing of late embryo mortality at D53. At birth, chicks (*n* = 109 from CT control and *n* = 118 from RU) were counted and weighted, sex was determined, and 20 chicks (10 CT (5 males and 5 females) and 10 RU (5 males and 5 females)) were slaughtered to collect and weigh subcutaneous fat and organs. At PND5 and PND10, food consumption was recorded along with body weight, and 20 chicks (10 CT (5 males and 5 females) and 10 RU (5 males and 5 females)) were slaughtered to weigh subcutaneous fat and different organs.

### 2.3. Treatment of Groups and Preparation of the Feed

Roosters were fed either Roundup (RU)-contaminated feed (*n* = 5) or control feed (*n* = 5) from the age of 32 to 37 weeks. Roundup (Gallup super 60) was obtained from Axereal (Monnaie, France); it contains 360 g/L glyphosate (485.8 g/L isopropylamine salt). The composition of the feed is indicated in Table 1.

The control group (CT) was fed with a feed containing low measurable glyphosate and AMPA concentrations (0.21 mg/kg feed for glyphosate and undetectable levels for AMPA as determined by Phytocontrol, Nimes, France). The concentration of glyphosate in the RU feed was 1250 mg/kg feed for glyphosate and 0.30 mg/kg feed for AMPA, as determined by Phytocontrol. Roosters (5.34 kg ± 0.1) were food-restricted as recommended by the provider, and their food consumption was 200 g/day. Thus, the concentration in the feed corresponded to a dose of 46.8 mg glyphosate/kg body weight/day in the roosters. The European Food Safety Authority (EFSA) has reported a NOAEL of 100 mg/kg body weight/day for poultry, with a maximum residue level (MRL) of 2.28 mg/kg bw/d [19]. Therefore, our experiment tested a concentration of approximately 47% of the NOAEL threshold.

### 2.4. Biological Samples

Blood samples from 10 32-week-old roosters were collected from the occipital sinus into heparin tubes at different days during the experiment (Days 0, 5, 13, 25 during RU exposure and Day 50, corresponding to 14 days after the end of RU exposure). Sperm was collected by manual stimulation from the same 10 adult roosters at the same time as the blood plasma. Sperm and blood samples were centrifuged (5000× *g* for 10 min at 4 °C) and stored at −20 °C for further investigations. Testis samples were obtained at different times of the experiment (D36, 36 days after the beginning of RU exposure and D50, 14 days after the end of RU exposure) by dissection after slaughtering. We chose 14 days after the end of RU exposure because this corresponds to one spermatogenesis cycle in roosters [26]. For each animal, one testis was stored at −80 °C for Western blot as well as testosterone and oestradiol assays and another one was fixed in a solution of 4% PAF overnight at room temperature for histological analysis.

### 2.5. Testosterone, Oestradiol and Chemerin Assays

The plasma and testis concentrations for testosterone and oestradiol as well as the seminal fluid chemerin concentrations were measured by Enzyme Linked Immunosorbent Assays (ELISA), using commercial kits (chicken testosterone: MBS703019 (sensitivity < 0.05 ng/mL, MyBioSource, San Diego, CA, USA), chicken oestradiol: MBS269416 (sensitivity: 2 pg/mL, MyBioSource, San Diego, CA, USA), chicken chemerin: MBS738819 (sensitivity 0.1 ng/mL, MyBioSource, San Diego, CA, USA)), according to the manufacturer’s instructions. All assays were performed in 96-well plates, and absorbance was measured at 450 nm using a microplate reader (Tecan, Magellan, Männedorf, Switzerland). A standard curve was drawn for the determination of hormone levels. The experiment was performed following the manufacturer’s protocol, with an intra-assay coefficient of variation < 15%.

### 2.6. Evaluation of Sperm Motility Using IVOS (Integrated Visual Optical System)

Semen from five CT and five RU roosters was individually analysed on each day of sperm collection. Computer-assisted sperm assessment (CASA) was performed using a Hamilton–Thorne motility analyser (Hamilton–Thorne Biosciences, Beverly, MA, USA) to determine total motility and the kinematic characteristics of sperm movement. The IVOS settings used were as follows: negative-phase contrast optics recording rate of 60 frames per second, minimum contrast of 50, minimum cell size of 4 pixels, cell size gate of 25 pixels and cell intensity of 80. Three microliters of sperm from each sample were placed into a pre-warmed (37 °C) 100-μm standard counting chamber (Makler^®^Counting Chamber, Clinisciences, Nanterre, France) before immediate transfer to IVOS. Sperm motility analysis was based on the four to five consecutive digitalised images obtained from a single field of view, acquired using a 10× negative-phase contrast objective. Images were taken with a time-lapse of 1 s, and objects incorrectly identified as sperm were removed from the analysis. The following motility parameters were evaluated: Percentage of motile sperm, VCL (curvilinear velocity in μm/s), VSL (straight-line velocity in μm/s), VAP (average path velocity, in μm/s), percentage of progressive motility and speed (μm/s). Parameter means were calculated by the average of summary values obtained from each sample. For each sample of sperm from five CT and 5 RU animals at different time points (Days 0, 5, 13, 25 and 50), 1000 spermatozoa were analysed at 37 °C in 100-μm standard counting chambers (Makler^®^ Counting Chamber, Clinisciences, Nanterre, France).

### 2.7. Determination of Adenosine Triphosphate (ATP) and Calcium Concentration in Spermatozoa

The ATP concentrations in sperm were measured using luciferin/luciferase reactions with Cell-Titer-Glo Assay (Promega, Madison, WI, USA). Standards were prepared from ATP standard (F203A, Promega) using serial dilutions to obtain concentrations of 1 × 10^−7^, 1 × 10^−8^, 1 × 10^−9^, 1 × 10^−10^, 1 × 10^−11^ and 1 × 10^−12^ M. Briefly, the assay buffer and substrate were equilibrated to room temperature, and the buffer was transferred to the substrate and gently mixed with it to obtain a homogeneous solution. After a 30-min equilibration of the cell plate to room temperature, 100 μL of sample and 100 μL of luciferin/luciferase reagent were added to the 96-well plates, the content was mixed for 2 min, and incubation was continued for 10 min at room temperature. Luminescence at integration time 1000 (ms) was read using an Ascent Luminoskan Luminometer (Thermo Scientific, Illkirch, France). For the determination of calcium concentrations in spermatozoa, CT or RU sperm suspensions (20 μL; final concentration 2 × 106 cells/mL) were centrifuged at 150× *g* for 15 min and lysed in RIPA buffer at 4 °C for 30 min, followed by sonication for 60 s on ice. The lysates were centrifuged at 10,000× *g* for 15 min, and Ca^2+^ concentrations were estimated in the supernatants using Arsenazo III (Sigma-Aldrich, Saint Quentin Fallavier, France) according to the method modified by Michaylova and Ilkova [27]. The intensity of the purple complex formed with the reagent was read at 600 nm in a spectrophotometer (Labtech LT-4000MS; Labtech International Ltd., Uckfield, UK), using the Manta PC analysis software. Protein concentrations were estimated in the pellets by the modified Lowry’s method [28]. The Ca^2+^ levels were calculated as mg/L. Both calcium and ATP concentration measurements were performed at four timepoints (Days 0, 13, 25 and 50) in sperm of five CT and five RU roosters.

### 2.8. Immunofluorescence

Spermatozoa were fixed with 4% PAF for 15 min at room temperature and deposited on a glass slide. Then, fixed spermatozoa were incubated with PBS 1X/0.1 M glycine for 15 min at room temperature to saturate the aldehyde groups and permeabilised with 0.1% Triton X-100 (*w*/*v*) in PBS for 15 min; nonspecific binding sites were blocked in 2% Bovine Serum Albumin (BSA)/PBS for 15 min. Cells were incubated for 60 min at room temperature with the primary monoclonal antibodies against DNA damage, diluted at 1:100 in 1% BSA/PBS (Sigma-Aldrich, l’Isle d’Abeau Chesnes, France); Mouse IgG (Sigma-Aldrich, l’Isle d’Abeau Chesnes, France), was used as negative control. After incubation, spermatozoa were washed three times in PBS and incubated for 45 min at room temperature with goat anti-mouse IgG Alexa Fluor^®^ 488 antibodies (diluted at 1:500 in 1% BSA/PBS). Subsequently, spermatozoa were counterstained with 4′,6′-diamidino-2-phenylindole (DAPI), mounted on glass slides with Fluoroshield mounting medium (Sigma-Aldrich, l’Isle d’Abeau Chesnes, France) and examined using standard immunofluorescence microscopy. Staining was quantified using the software Image J (NIH, Bethesda, MD, USA) on at least 500 spermatozoa per animal (*n* = 2 CT and 2 RU at the end of RU exposure and *n* = 3 CT and *n* = 3 RU at 14 days after RU exposure).

### 2.9. Histological Examination of the Testes

Testes embedded in paraffin were serially sectioned to a slice thickness of 7 μm. Deparaffinised sections were hydrated and washed in a PBS bath for 5 min and subsequently stained with a haematoxylin-eosin solution (Sigma-Aldrich, l’Isle d’Abeau Chesnes, France). The diameter of the round or nearly round transverse section of the seminiferous tubule was measured for each testis using the software ImageJ (NIH, Bethesda, MD, USA) (*n* = 30 measurements per animal, *n* = 2 CT and *n* = 2 RU animals at the end of RU exposure and *n* = 3 CT and *n* = 3 RU animals 14 days after RU exposure).

### 2.10. Fertility Parameters

Forty 32-week-old hens were divided into 10 pens, each containing four hens. Twenty hens (5 pens) were artificially inseminated with a pool of 200 million spermatozoa obtained from CT roosters and the other 20 hens (5 pens) were artificially inseminated with a pool of 200 million spermatozoa obtained from RU roosters. Each hen was inseminated twice at an interval of 2 days. Eggs were collected the day after the last day of AI for 7 days and then artificially incubated. We assessed the number of unfertilised eggs, early (EEM) and late (LEM) embryonic mortality by breaking eggs and candling on the 7th (EEM) and 14th (LEM) days of incubation, respectively, as described in Barbe et al. (2020) [29]. The different percentages (EEM, LEM, hatchability of fertile eggs and fertility) were calculated using the following equations:

%EEM = number of EEM/(number of incubated eggs-unfertilised eggs) × 100;

%LEM = number of LEM/(number of incubated eggs-(unfertilised eggs +number of EEM)) × 100;

%Hatchability of fertile eggs = (number of hatched chicks/number of fertile eggs after 14 days of incubation) × 100;

%Fertility = (number of fertile eggs after 14 days of incubation/number of incubated eggs) × 100.

### 2.11. Glyphosate and AMPA Assays in Seminal Liquid and Plasma

Glyphosate and AMPA were measured in blood and seminal plasma of roosters after a derivatisation reaction using FMOC-Cl (9-fluorenylmethyl chloroformate), in collaboration with Dr S El Balkhi (Service de Pharmacologie, Toxicologie et Pharmacovigilance, Limoges, France). Samples were extracted with diethyl ether and injected into the LC/MS-MS system. Glyphosate 13C215N was used as an international standard and purchased as a solution of 100 mg/L (LGC, UK); prior to use, it was diluted in deionised water to obtain a working solution of 0.5 mg/L. Glyphosate and AMPA (LGC, UK) were of 98.69 and 99% purity, respectively, and were dissolved in deionised water to obtain working solutions at increasing concentrations, ranging from 0.01 to 50 mg/L. These standard solutions were used to spike glyphosate-free urine for the preparation of the calibration curves for standards. Six calibration standards between the higher limit of quantification (LOQ) and the lower LOQ (namely between 0.1 and 10 µg/L) were necessary for the calibration. The FMOC (Acros Organics, Belgium) was prepared at 50 g/L and used for the derivatisation reaction. Glyphosate and AMPA already derivatised with FMOC were purchased from LGC (98 and 99.6% purity, respectively). Working solutions of glyphosate-FMOC and AMAP-FOMC at 0.1 and 1 mg/L were used to spike glyphosate-free urine samples to prepare internal quality controls at 0.5 and 5 µg/L. A 50-µL volume of IS and 1 mL of 0.5 M tetraborate buffer (pH 9) were added to 1 mL of blood or seminal plasma. Then, 3 mL of the FMOC solution was added, and the sample was allowed to stand for 30 min in the dark. For the extraction of the formed derivatives, 1 mL of 6M HCl and 6 mL of diethyl ether were added to each sample, followed by agitation for 15 min and centrifugation at 3000× *g* for 5 min. The organic phase was then transferred to a 15-mL glass tube and evaporated to dryness under nitrogen flow. The dried sample was taken up in 200 µL of (50/50) mobile phase solutions, and a 10-µL aliquot was injected into the LC-MS/MS system. The calibration standards were treated in the same way after spiking of the appropriate volume of the working solutions.

The LC-MS/MS system included a Shimadzu NEXERA X2 series and an 8060 triple quadrupole mass spectrometer. Chromatographic separations were performed at 40 °C on a Kinetex C18 100A column (100 × 2.10 mm, 2.6 µm particles) (Phenomenex, France). Mobile phase A contained 0.05% formic acid, and phase B included acetonitrile and 0.05% formic acid. Identification and quantification of glyphosate-FMOC and AMPA-FMOC were performed in negative mode using the MRM of a quantifier ion (390.2/62.9 and 331.9/110.1, respectively) and an additional qualifier ion (389.9/168.1 and 331.9/62.9, respectively). To meet the criteria for positive identification, the ratio between the quantitative and the qualifying transition ions (derived from the precursor ion) had to fall within ±20% of that established by the calibration standards.

### 2.12. Western Blot

Proteins were extracted from the testes of CT and RU roosters on D36 and D50 in lysis buffer (Tris 1 M (pH 7.4), NaCl 0.15 M, EDTA 1.3 mM, EGTA 1 mM, VO43−23 mM, NaF 0.1 M, NH2PO41%, Triton 0.5%), using an Ultraturax (Invitrogen ™ by Life Technologies ™, Villebon-sur-Yvette, France) as previously described [30]. The lysates were centrifuged for 20 min at 16,000× *g* and 4 °C, and the supernatants containing proteins were collected and kept on ice. Protein concentrations were measured using the bicinchoninic acid (BCA) protein assay (Interchim, Montluçon, France). Lysates (80 μg) were mixed with Laemmli buffer 5×, and proteins were denatured for 5 min at 95 °C. Subsequently, proteins were loaded in an electrophoresis sodium dodecyl sulphate-polyacrylamide gel (12%) and transferred onto a nitrocellulose membrane. Membranes were blocked with Tris-Buffered Saline Tween buffer containing 0.05% Tween 20 and 5% skimmed milk for 30 min at room temperature. Membranes were then incubated overnight at 4 °C with the appropriate primary antibodies (3beta-hydroxysteroid-dehydrogenase (ThermoFisher Scientific, Illkirch-Graffenstaden, France, reference PA5-106895), Cytochrome P450 11a1 (P450scc, ThermoFisher Scientific, reference PA5-37359), Steroidogenic acute regulatory (StAR, ThermoFisher Scientific, reference PA5-21687) and vinculin (Sigma, Saint Quentin Fallavier, France, reference V9131)) at a dilution of 1/1000. Then, membranes were incubated for 90 min at room temperature with horse radish peroxidase-conjugated anti-rabbit or anti-mouse antibodies (Bio-Rad Laboratories, Marnes-la-coquette, France) at a dilution of 1/5000. Proteins of interest were detected by enhanced chemiluminescence (Western Lightning Plus-ECL, Perkin Elmer, Villebon-sur-Yvette, France) with the G-box SynGene (Ozyme, St Quentin en Yvelines, France) and GeneSnap software (Ozyme, St Quentin en Yvelines, France). Subsequently, proteins were quantified with the GeneTools software (version 4.01.02; Syngene). The results were expressed as the intensity signals in arbitrary units after normalisation with vinculin.

### 2.13. Determination of Mortality, Food Consumption, Body and Different Organ Weights in Offspring

The chicks (*n* = 109 and 118 chicks from CT and RU roosters, respectively) were weighted at hatching (Day 0 or PND0) as well as 5 and 10 days of age (Day 5 or PND5 and Day 10 or PND10). At hatching, chicks were divided into 10 pens (5 pens for 109 chicks from CT roosters and 5 pens for 118 chicks from RU roosters). Each pen had almost the same number of male and female chicks. All animals were fed ad libitum with the same starting diet without RU exposure. The amount of food was recorded at Days 5 and 10 for each pen. Each day, the number of dead animals was recorded, and the mortality level was calculated from hatching to Day 10. At hatching as well as Days 5 and 10, 10 chicks (5 males and 5 females) from each group (from CT and RU roosters) were killed, and their organs or tissues (subcutaneous adipose tissue, brain, heart, liver and digestive tract) were dissected and weighted.

### 2.14. Oxidative Stress in Spermatozoa

The ROS-Glo™ H_2_O_2_ Assay (Promega, Charbonnieres, France) was used to analyse oxidative stress in spermatozoa. Assays were performed according to the manufacturer’s instructions. Briefly, after treatment, samples were stressed with H_2_O_2_ substrate solution for 3 h and incubated for 20 min with ROS-Glo™ Detection Solution in the dark to stabilise the luminescent signal. The plate was measured using a Luminoskan Ascent microplate reader (VWR International, Fontenay-sous-Bois, France) to record luminescence.

### 2.15. Analysis of Genomic DNA Methylation of the CT and RU Spermatozoa

Quantification of 5-methylcytosine (5mC) was performed on CT and RU spermatozoa samples. Genomic DNA was extracted with incubation for 4 h in a lysis buffer (10 mM Tris pH 8.0, 0.1 mM EDTA, 150 mM NaCl, 1% SDS) containing proteinase K (10 mg/mL). The QuiAMP DNA mini kit (Qiagen, Germany) was used for genomic DNA purification and the 5mC DNA ELISA kit (Enzo Life Sciences, Villeurbanne, France) for the quantification of 5mC using 100 ng of genomic DNA.

### 2.16. Statistical Analysis

All statistical analyses were performed using GraphPad Prism 6^®^ (San Diego, CA, USA). Data are presented as mean ± SEM. The means of independent and random replicates were used. Bartlett’s test was run to test the homogeneity of variance, and normal distribution was verified by the Shapiro–Wilk test. For parametric values, unpaired *t*-test and two-way ANOVA test, followed by a Sidak multiple comparison test, were applied as appropriate. For non-parametric values (unequal variances), we used the Mann–Whitney test, followed by Dunn’s multiple comparison test. Means were considered different at *p* < 0.05.

## 3. Results

### 3.1. Measurement of Glyphosate (G) and AMPA Concentrations in the Blood Plasma (BP) and the Seminal Fluid (SF) of Roosters

**Figure 2:**
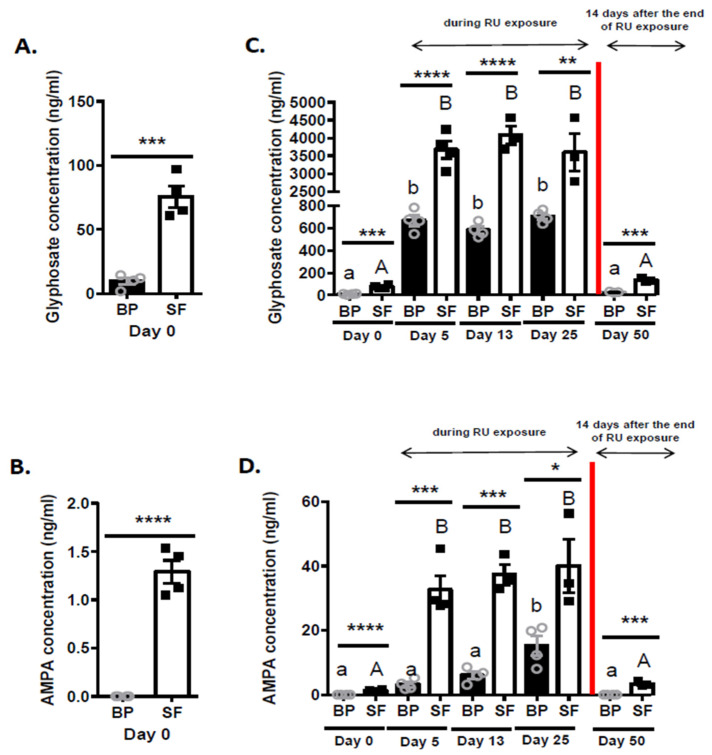
Determination of glyphosate and AMPA concentrations in the blood plasma (BP) and the seminal fluid (SF) in CT (*n* = 5) and RU roosters (*n* = 5). (**A**) Glyphosate assay in the BP and the SF at Day 0 (ng/mL). (**B**) AMPA assay in the BP and the SF at Day 0 (ng/mL). (**C**) Glyphosate assay in the BP and the SF at different times of the experiment (ng/mL). (**D**) AMPA assay in the BP and the SF at different times of the experiment (ng/mL). Lowercase letters correspond to the ordinary one-way ANOVA significance (*p* < 0.05) for BP analysis and uppercase letters correspond to the ordinary one-way ANOVA significance (*p* < 0.05) for SF analysis, followed by Tukey’s multiple comparisons test comparing the evolution of the concentration of glyphosate or AMPA in both compartments at different days; Stars (*) correspond to the unpaired *t*-test significance (*p* < 0.05) corresponding to the comparison between BP and the SF compartment. ** *p* < 0.01; *** *p* < 0.001; **** *p* < 0.0001.

Glyphosate concentrations were about 7-fold higher in SF than in BP in control roosters at Day 0 (no dietary G exposure, 9.66 ± 2.77 ng/mL vs. 75.37 ± 8.16 ng/mL, *p* = 0.0003 Figure 2A). In the same animals, AMPA was undetectable in BP, whereas its concentrations were 1.29 ± 0.12 ng/mL in SF (Figure 2B). Similar data were observed at Day 5, 13, 25 and 50 (Figure S1A,B). After 5, 13 and 25 days of dietary RU exposure, the G concentrations increased as compared to Day 0 (no exposure) by 69 (*p* < 0.01), 60 (*p* < 0.01) and 73 times (*p* < 0.01) in BP and by 49 (*p* < 0.01), 54 (*p* < 0.01) and 47 times (*p* < 0.01) in SF, respectively (Figure 2C). During this same period, AMPA concentrations were 3 (*p* < 0.01), 6 (*p* < 0.01) and 15 times -fold (*p* < 0.01) times higher in BP and by 33 (*p* < 0.01), 37 (*p* < 0.01) and 40 times (*p* < 0.01) in SF, respectively (Figure 2D). For each period of RU exposure, glyphosate and AMPA concentrations were significantly higher in SF than in BP (Figure 2C,D). Fourteen days after exposure to RU had ceased, both glyphosate and AMPA concentrations were significantly reduced and returned close to the basal state (Day 0) (Figure 2C,D).

### 3.2. Evaluation of Chronic RU Dietary Exposure on Sperm Concentration and Motility in Roosters

**Figure 3:**
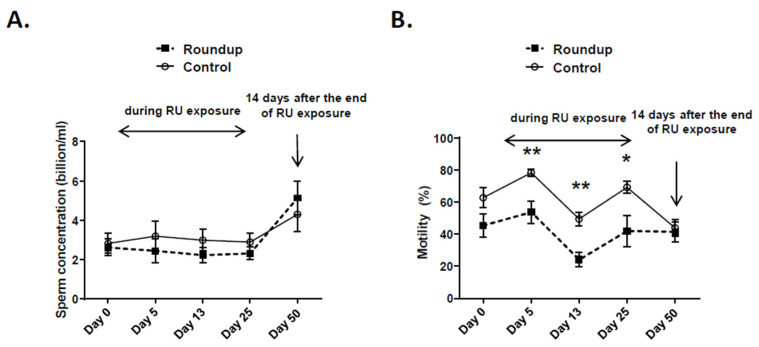
Sperm parameters in CT and RU roosters. (**A**) Measurement of the sperm concentration at different times of the experiment (billion/mL). (**B**) Assessment of the percentage of sperm motility at different times of the experiment. Stars (*) correspond to the unpaired *t*-test significance (*p* < 0.05), ** *p* < 0.01. CT: control; RU: Roundup.

Dietary RU exposure had no significant effect on sperm concentrations, irrespective of the exposure time (Figure 3A), whereas it significantly reduced the percentage of motility after 5, 13 or 25 days of dietary exposure (*p* < 0.05, Figure 3B) within the RU group. However, this inhibitory effect ceased 14 days after the end of exposure (Day 50, Figure 3B).

Dietary RU exposure significantly reduced the percentage of progressive motility, the speed of spermatozoa and the VSL, irrespective of RU exposure duration, and the VAP at Days 5 and 25 as well as VCL at Day 25 only; these deleterious effects ceased at Day 50 (Table 2).

**Figure 4:**
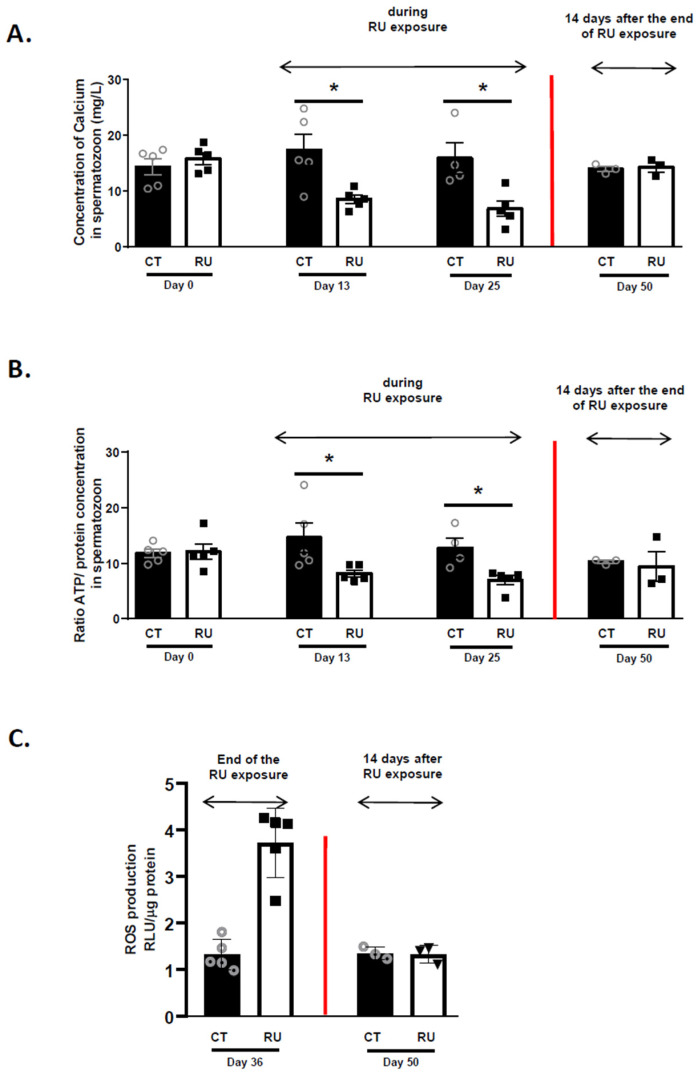
ATP and Ca^2+^ quantification and ROS content in CT and RU spermatozoa. (**A**) Ca^2+^ assay in spermatozoa of CT (*n* = 5) and RU (*n* = 5) roosters at Days 0, 13, 25 and 50 (14 days after the end of RU exposure (*n* = 3 CT and *n*= 3 RU roosters) (mg/L). Stars (*) correspond to the unpaired *t*-test significance (*p* < 0.05). (**B**) Ratio between ATP concentration and protein concentration in spermatozoa of CT and RU roosters at Days 0, 13, 25 (*n* = 5 CT and *n* = 5 RU roosters at each time) and 50 (*n* = 3 CT and *n* = 3 RU roosters 14 days after the end of RU exposure). (**C**) Reactive Oxygen Species (ROS, H_2_0_2_) levels in spermatozoa (*n* = 5 CT and 5 RU animals at Day 36 and *n* = 3 CT and *n* = 3 RU animals at Day 50). Stars (*) correspond to the unpaired *t*-test significance (*p* < 0.05).

Dietary RU exposure halved calcium and ATP concentration in spermatozoa after 13 and 25 days of exposure, respectively (Figure 4A,B). In addition, we measured a higher ROS content in spermatozoa after 36 days of exposure (Figure 4C). Again, these negative effects were no longer observed at Day 50 (Figure 4A,B). Furthermore, after slaughter of a few animals, we did not observe any difference in the weight of the testes of the animals, whether after 36 days of exposure (38 ± 3 g CT *n* = 2; 27.5 ± 2.5 g RU, *n* = 2) or 14 days after the end of RU exposure (Day 50, 40.3 ± 1.3 g CT *n* = 3; 40 ± 1.5 g RU, *n* = 3). Testicular histology also revealed that the diameter of the seminiferous tubules was reduced in the RU group on D36 and D50 as compared to CT animals (Figure S2A,B). In addition, sperm quality was affected on D36 since spermatozoa from RU animals had a more abnormal head morphology (Figure S3A,B) but also more DNA damage (Figure S3C). These latter effects were reduced or totally ceased on D50 when animals had not been exposed for a period of 2 weeks.

### 3.3. Effect of Roundup Dietary Exposure on Plasma Steroid Concentration in Roosters

**Figure 5:**
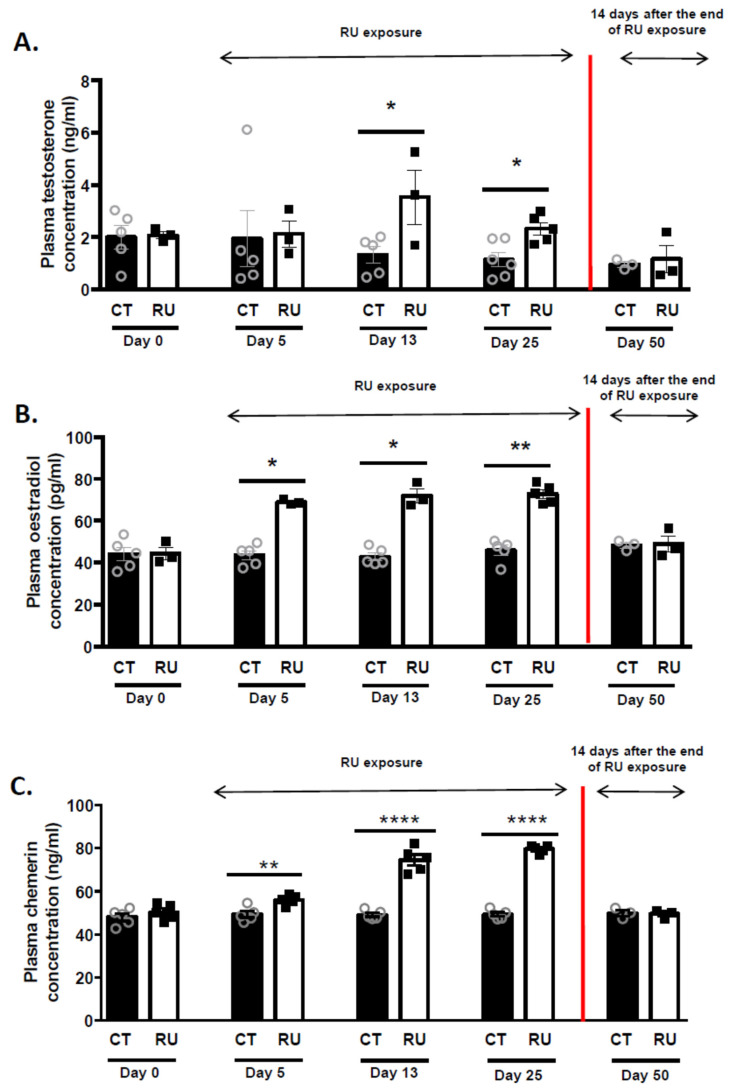
Plasma testosterone, oestradiol and chemerin concentrations in CT and RU animals. (**A**) Concentration of blood plasma testosterone (ng/mL). Stars (*) correspond to the unpaired *t*-test significance (*p* < 0.05) between CT and RU rooster groups at different times. (**B**) Concentration of plasma oestradiol (pg/mL). Stars (*) correspond to the unpaired *t*-test significance (*p* < 0.05) between CT and RU rooster groups at different times. ** *p* < 0.01. (**C**) Concentration of plasma chemerin (ng/mL). Stars (*) correspond to the unpaired *t*-test significance (*p* < 0.01) between CT and RU rooster groups at different times. ** *p* < 0.01; **** *p* < 0.0001.

Plasma testosterone concentrations were significantly increased after 13 (*p* < 0.05) and 25 (*p* < 0.05) days of dietary RU exposure, as shown in Figure 5A. Similar data were observed for plasma oestradiol concentrations, with a significant effect as soon as after 5 days of exposure (*p* < 0.05, Figure 5B). This increase in plasma steroid concentrations in response to dietary RU exposure ceased 14 days after ending dietary exposure (D48). The SF chemerin concentrations were significantly higher in RU animals as compared to control animals (CT), irrespective of the exposure period (5, 13 and 25 days, Figure 5C). After slaughter of a few animals, we collected testes and showed that testis testosterone and oestradiol concentrations increased in RU as compared to CT animals at Day 36 but not at Day 50 (Figure S4). In addition, at Day 36, the protein level of the cholesterol side-chain cleavage enzyme (P450scc) and the cholesterol level in the testes was higher in RU animals that in control animals, whereas the 3-beta–hydroxysteroid dehydrogenase (3βHSD) level and the amount of the cholesterol carrier, steroidogenic acute regulatory protein (STAR), was similar in both groups (Figure S4). This positive effect of dietary RU exposure was no longer observed at D50 (Figure S4).

### 3.4. Effect of Roundup Dietary Exposure on In Vivo Fertility

Furthermore, we investigated whether the negative effect of dietary RU exposure on sperm motility could affect in vivo fertility. The percentages of unfertilised eggs, early (EEM) and late embryonic mortality (LEM), hatchability of fertile eggs and fertility are shown in Table 3. No significant difference was observed between the CT and RU groups for all these fertility parameters.

### 3.5. Effect of Paternal Chronic Dietary Roundup Exposure on Mortality, the Food Intake, Growth and Fattening of the Progeny

**Figure 6:**
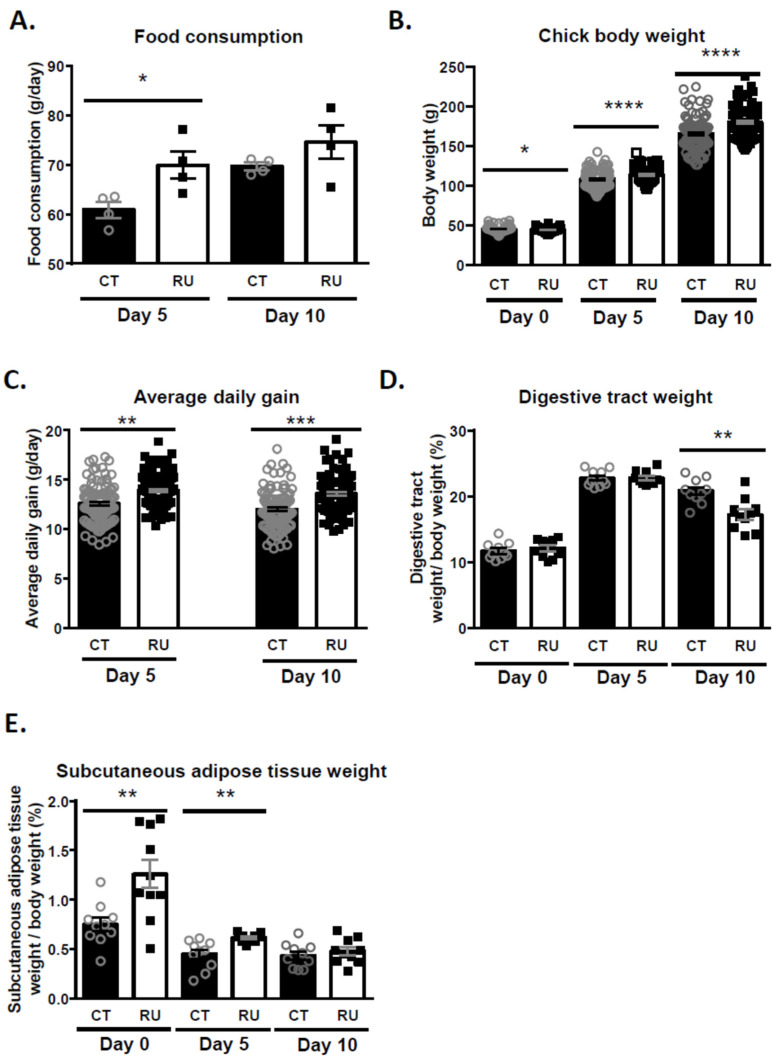
Consequences of RU exposure on the metabolism of offspring. (**A**) Analysis of RU and CT chicks’ food consumption at postnatal Days (PND) 5 and 10. Average food consumption per pen (g/day) was determined. Stars (*) correspond to the unpaired *t*-test significance (*p* < 0.05). (**B**) Body weight (g) of RU (*n* = 118) and CT chicks (*n* = 109) at birth and on PND 5 and 10 (g). Stars (*) correspond to the unpaired *t*-test significance (*p* < 0.05). **** *p* < 0.0001. (**C**) Average daily gain (g/day) for CT (*n* = 109) and RU chicks (*n* = 118) on PND 5 and 10. Stars (**) correspond to the unpaired *t*-test significance (*p* < 0.01), *** *p* < 0.001. (**D**) Evaluation of the ratio between the digestive tract weight and the body weight of CT chicks (*n* = 10) and RU chicks (*n* = 10) at birth and on PND 5 and 10 (%). Stars (*) correspond to the unpaired *t*-test significance (*p* < 0.05). (**E**) Evaluation of the ratio between subcutaneous adipose tissue weight and body weight of CT chicks (*n* = 10, 5 males and 5 females) and RU chicks (*n* = 10, 5 males and 5 females) at birth and on PND 5 and 10. Stars (**) correspond to the unpaired *t*-test significance (*p* < 0.01).

We next assessed the mortality level between hatching (Day 0) and 10 days (Day 10), the food consumption at Days 5 and 10, the body weight, the average daily gain and the weights of various tissues (liver, brain, heart, digestive tract and subcutaneous adipose tissue) at Days 0, 5 and 10 of chicks from the two groups of fathers (CT and RU) (Figure 6). The percentage of mortality was not significantly different between CT (2.1 ± 0.2%) and RU (2.2 ± 0.3%) chicks. Food consumption (Figure 6A), body weight (Figure 6B) and average daily gain of chicks (Figure 6C) from fathers exposed to RU (RU group) were significantly higher than those of chicks from control fathers (not exposed to Roundup: CT group). These data were observed at hatching (D0), 5 (D5) and 10 days (D10) of age (except for food consumption at D10). The ratio between the digestive tract weight and the total body weight was significantly reduced in RU compared to CT chicks at 10 days of age (*p* < 0.01) (Figure 6D). In contrast, the ratio between subcutaneous adipose tissue weight and total body weight was significantly higher at D0 and D5 (*p* < 0.01) in RU than in CT animals (Figure 6E). No difference in terms of liver, heart and brain weight was observed in chicks from the two groups of roosters. No significant difference was observed between the CT and RU group for all these fertility parameters.

### 3.6. Analysis of Genomic DNA Methylation of CT and RU Spermatozoa

**Figure 7:**
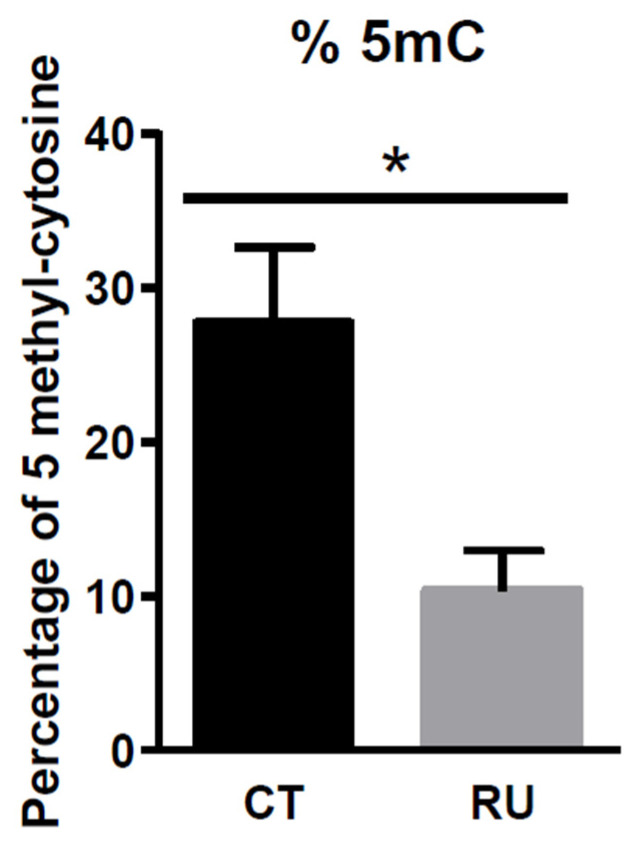
Methylation of DNA spermatozoa from CT and RU roosters during RU exposure. Percentage of 5 methylcytosine in the DNA of spermatozoa of CT (*n* = 5) and RU (*n* = 5) roosters at Day 25 (25 days after RU exposure). Stars (*) correspond to the unpaired *t*-test significance (*p* < 0.05).

Our previous data about progeny led us to investigate the 5mC profile of the spermatozoa genomic DNA from RU or CT roosters. We observed hypomethylation in the RU roosters’ spermatozoa DNA (*p* < 0.05) when the animals were exposed to dietary RU for 36 days (Figure 7). Similar data were found when animals were exposed for 25 days.

## 4. Discussion

Our results indicate that dietary paternal RU exposure in roosters leads to a significant reduction in sperm motility, an increase in steroidogenesis and a higher food consumption, growth performance and fattening in the progeny. However, we detected no effect on fertility and embryo mortality. We also showed, for the first time, the presence of higher glyphosate and AMPA concentrations in the seminal fluid as compared to blood plasma.

Here, dietary RU exposure is the equivalent of 46.8 mg/kg body weight/day of glyphosate, which is half of the NOAEL in birds. However, EFSA, in their report, specified that the study run on chickens had some limitations, and therefore, the NOAEL was established for birds [19]. The concentration of glyphosate in the RU diet was 1250 mg/kg feed, which is about 3 times higher than the amounts found in grains after GBHs are spread on a grain field [31]. Non-ionic surfactants, such as polyoxyethylene amine (POEA), are also included in the formulas of many herbicides and act by increasing the capacity of the active ingredients to penetrate the leaf cuticle [32]. Some studies indicate that compounds containing surfactants may be more toxic than glyphosate alone [33] and may persist in the soil for 2 or more years [34]. Thus, we chose to determine the effect of RU and not that of G alone. We first assessed the G and AMPA concentrations in blood and seminal plasmas in roosters. Interestingly, we found a low G concentration before dietary RU exposure, ranging from 3–9 µg/L, similar to the concentrations found in the urine of humans not exposed to GBHs [4] and to those in the maternal serum of Thai women [35]. In response to dietary RU exposure, the G concentration increased in both compartments (blood and seminal plasma) in a time-dependent manner, suggesting an accumulation. An in vivo study on humans determined that after consuming food with a known amount of G (192 µg) and AMPA, only 1% of G and 23% of AMPA were found in the urine; the half-life of G was around 9 h [36]. In humans, G seems to accumulate principally in the kidneys, liver, colon and small intestine and is eliminated via the faeces (90%) and urine within 48 h [37]. In the present study, the amount of G was more important in the seminal plasma than in the blood plasma. It remains to investigate how and why G and AMPA are accumulated in the seminal plasma. No AMPA was detected prior to exposure in the blood plasma. For both G and AMPA, the level observed at D0 was recovered at D50, 14 days after the end of exposure to RU. This is in accordance with the half-life of G which is about 5–10 h [38].

In our study, sperm motility was reduced at D5, D13 and D25 after RU exposure. These data are in good agreement with those observed in vivo in rats [39,40,41], mice [8], and pigs [42,43] and in vitro in humans [44,45]. In addition, the calcium and ATP concentrations in spermatozoa were significantly reduced in RU animals. These two parameters are involved in the flagellar movement [46]. Calcium metabolism is also linked to the variation of sperm velocity [47]. Interestingly, increased calcium levels were observed in in vitro prepubertal rat testes after exposure to RU for 30 min (720 µg/L–360 mg/L) [48]. The discrepancy with the present study could be explained by RU exposure (acute vs chronic), the species (rat vs. rooster) and the type of the experiment (in vitro vs. in vivo). Thus, the lower calcium and ATP concentrations in spermatozoa from RU animals could explain their lower motility. Several studies have shown a negative role of one adipokine named chemerin in the regulation of the male reproductive system [30,49]. In humans, the level of chemerin is higher in the blood than in seminal fluid [49]. A higher concentration of chemerin in the seminal plasma is associated with a lower sperm quality and fertility in roosters [30]. Here, we observed that the level of chemerin was higher in the seminal fluid of RU roosters than in that of the CT group. Hence, the reduction of the percentage of sperm motility in RU animals could be related to the increased level of chemerin in the seminal fluid. No impact on sperm concentration was recorded after dietary RU exposure, which contrasts with previous findings. Indeed, an exposure to G (500 mg/kg bw/d) for 5 weeks [50] or to RU (50 mg/kg bw/d) during the gestational period [9] or during adulthood in rats [40], mice [8], pigs [42] and in rabbits [51] led to a reduced sperm concentration. However, the effect of RU or G on the sperm concentration could be dependent on the species but also on the timing of exposure. In the present study, we also observed a few animals in which the percentage of sperm with abnormal head morphology was significantly higher in RU than in CT roosters. In previous studies, exposure to G or RU during adulthood led to an increased rate of abnormal sperm in rats [39,40,52], mice [8], pigs [42] and rabbits [51]. However, until now, no data were available on the effect of GBHs on avian spermatozoa. In a few animals, we also observed that a dietary RU exposure did not affect the weight of the testis in roosters but significantly decreased the diameter of the seminiferous tubules; this effect was maintained for 2 weeks after the end of RU exposure. Modifications of seminiferous tubules morphology have been described after RU exposure in rats [53]. Moreover, Liu et al. (2022) [54] reported an association between gut microbiota alteration and defective spermatogenesis in rats exposed to G (50 mg/kg bw/d) by food. The authors demonstrated that gut microbiota dysbiosis-driven local Interleukin-17A (IL-17A) production could be responsible for male reproductive toxicity induced by G. In the present study, dietary RU exposure could provoke the decrease in sperm motility though the activation of Th17 cells and the increase in IL-17A production and, consequently, the increase in testis inflammation. However, more analyses must be performed to validate these hypotheses.

Furthermore, we noted an increase in plasma testosterone and oestradiol concentrations in RU-exposed roosters. These effects could be explained by an increase in cholesterol level and expression of the steroidogenesis enzyme P450scc in testis. Our data are in good agreement with two others studies performed in rats, where the level of testosterone increased after RU exposure during the gestational period to weaning in the serum [53] and in the testes [7]. However, other studies described that RU exposure decreased plasma testosterone levels in rodents [9,11,12,55] and Japanese quails [20]. Here, plasma oestradiol was also increased by RU exposure at D5, 13 and 25 as well as in the testes at D36. This is in accordance with studies conducted on rats [53,56]. We also observed no significant effect of paternal dietary RU exposure on the fertility parameters. This result could be explained by the great number of spermatozoa used to inseminate the hens. Ruuskanen et al. (2020) [20,21] showed that parental exposure to RU for 12 months in female quails did not change egg quality (egg, yolk, shell mass); however, there was a tendency to poor embryo development. Moreover, in ovo injection of G (10 mg/kg egg mass of pure G) or RU (G equivalent) decreased the percentage of hatching [24].

Here, we show for the first time that chicks from dietary RU-exposed fathers had a higher food intake, body weight and average daily gain during the first 10 days. This was not observed in in ovo studies in chickens [24,25], and the opposite was observed in in vivo studies in the F1 generation [14,57] or the F2 generation [58]. In the literature, the opposite effect was also observed in F1 adult mice [59] when they were pre- and post-natally exposed to GBH. In addition, we noted a higher amount of subcutaneous adipose tissue mass, which is one of the most susceptible organs to be affected by an endocrine disruptor, including RU [60]. Fathi et al. [24,25] observed an increase in the liver mass in hatched chicks after in ovo exposure to RU. Another study on chicken embryos exposed in ovo to RU found a reduction in the heart and liver mass on embryonic Day 18 [61]. In female mice exposed to G for 20 weeks (2 mg/kg bw/d), no perturbation of the weight of various organs (heart, liver, spleen, kidneys and uterus) was observed [62]. Thus, the effects of G on the offspring are dependent on the injection pathway (alone or combined with formulants) and if they are transmitted through the mother or directly in ovo injected. In recent studies [38,63], epigenetic analyses were run to understand how pesticides can impact the regulation of genes in the long term and how they can affect the next generation. The DNA methyltransferase gene (Dmnt1) enables keeping the same methylation pattern during DNA replication and cell division, and Dmnt3 adds methylation de novo [64].

In medaka embryos, RU exposure for the first 15 days of embryonic life induced a reduction of Dmnt1 mRNA amounts in the testes, leading to a global hypomethylation [64]. Thus, in good agreement with the literature, we observed a global hypomethylation of the genes in the spermatozoa of RU roosters (F0 generation) compared to the CT group. It could be interesting to analyse the impact of this change on the next generations (F1, F2, F3), since the most important consequences appear in the F3 generation, which is indirectly exposed to GBHs [38,63]. Moreover, in rats, G had a cytostatic effect and could interact with regulators of the cell cycle, such as eEF1α1, leading to a decrease in cell proliferation and a delay in protein synthesis, with a consequent suppression of spermatogenesis [16]. In the present study, the spermatozoa concentration was not affected, whereas the sperm motility was reduced, and the body weight of the offspring increased. These data suggest that dietary RU exposure in fathers did not negatively affect protein synthesis in the progeny, at least at the first week of age.

## 5. Conclusions

Here, we show for the first time that dietary RU exposure in roosters at a concentration half of that of the NOAEL significantly increased the G and AMPA concentrations in seminal plasma as compared to blood plasma. This great amount of G and AMPA in seminal plasma was associated with a lower motility of spermatozoa as well as a global hypomethylation of their DNA, without affecting sperm concentration. Higher plasma testosterone and oestradiol levels were also noted in roosters exposed to RU. Finally, dietary RU exposure in roosters did not affect male fertility and embryo development in our experimental conditions. However, it significantly increased food intake and average daily gain and the amount of subcutaneous adipose tissue in the progeny, suggesting epigenetic modifications. Thus, more experiments need to be performed to redefine or not the NOAEL of glyphosate for poultry.

## Figures and Tables

**Table 1:** Composition (%) of the diet.

Ingredient	%
Corn	39.64
Wheat	23.86
Soybean meal	29.3
Soy oil	2.2
Sunflower oil	1
Sodium bicarbonate	0.18
Calcium carbonate	0.86
Phosphate	2.06
Salt	0.3
Methionine DL	0.1
Mineral premix	0.5

**Table 2:** Effects of dietary Roundup exposure on sperm parameters in fresh rooster semen.

Sperm Parameters	Days	CT	SEM	RU	SEM
VAP (μm/s)	Day 0	60.3	±3.2	60.7	±2.3
	Day 5	74.4	±1.0	66.5 *	±3.3
	Day 13	61.2	±2.3	56.8	±2.2
	Day 25	71.8	±4.1	61.6 *	±2.2
	Day 50	65.9	±3.6	64.6	±1.0
VSL (μm/s)	Day 0	39.4	±3.5	40.5	±2.2
	Day 5	55.1	±1.3	46.6 *	±3.4
	Day 13	40.5	±1.6	34.1 *	±2.4
	Day 25	51.7	±3.3	39.7 ***	±2.1
	Day 50	44.6	±4.1	43.0	±1.1
VCL (μm/s)	Day 0	133.5	±3.0	136.8	±4.0
	Day 5	140.9	±1.8	136.7	±3.9
	Day 13	133.0	±3.1	125.6	±2.5
	Day 25	142.4	±3.4	131.8 *	±2.9
	Day 50	129.5	±5.7	129.3	±0.9
Progressive motility (%)	Day 0	15.6	±3.4	11.8	±2.4
	Day 5	35.3	±2.3	15.4 **	±4.5
	Day 13	12.2	±1.8	3.9 **	±0.8
	Day 25	24.9	±3.5	9.6 *	±3.4
	Day 50	14.3	±3.5	12.1	±3.0
Speed (μm/s)	Day 0	43.1	±6.2	31.3	±5.0
	Day 5	63.7	±2.6	33.9 ***	±6.6
	Day 13	31.0	±2.9	15.3 ***	±2.1
	Day 25	52.4	±4.2	26.9 *	±7.2
	Day 50	32.1	±4.0	30.3	±4.5

Average path velocity (VAP); straight-line velocity (VSL); curvilinear velocity (VCL); percentage of progressive motility and speed. Data are represented as mean ± SEM. Stars (*) correspond to the unpaired *t*-test (* *p* < 0.05; ** *p* < 0.01; *** *p* < 0.01).

**Table 3:** Percentages of unfertilised eggs, early (EEM) and late (LEM) embryonic mortality and fertility after artificial insemination in hens with sperm from RU (dietary exposure to Roundup) and control (CT) roosters. Results are presented as means ± SEM.

Parameters	Sperm Pool from 5 CT Roosters	Sperm Pool from 5 RU Roosters	*p*-Value
Unfertilised	6.75 ± 0.89	6.75 ± 0.75	0.90
EEM	2.51 ± 1.04	1.51 ± 0.92	0.44
LEM	0.80 ± 0.80	0.83 ± 0.83	0.99
Hatchability of fertile eggs	90.87 ± 3.40	91.11 ± 2.37	0.99
Fertility	93.98 ± 3.42	93.28 ± 2.10	0.81

## Data Availability

All the data will be available.

## Supplementary Materials

The following are available online at https://www.mdpi.com/article/10.3390/toxics9120318/s1, Figure S1. (A) Glyphosate and (B) AMPA concentrations (ng/mL) in the BP and the SF at Day 0, 5, 13, 25 and 50 in control roosters (*n* = 5). Stars (*) correspond to the unpaired *t*-test significance (*p* < 0.05) corresponding to the comparison between BP and the SF compartment. **** *p* < 0.0001. Figure S2. Morphology of testis and from CT and RU roosters. (A) Pictures of testis from CT and RU roosters at Day 36 (at the end of RU exposure) and Day 50 (14 days after RU exposure) with a light microscope, magnification ×10 and ×40 after staining with haematoxylin. (B) Measurement of the diameter (μm) of the seminiferous tubules of the testes of CT and RU roosters at Day 36 (*n* = 4 testes from 2 CT and 2 RU animals) and Day 50 (*n* = 6 testes from 3 CT and 3 RU animals). Stars (*) correspond to the unpaired *t*-test significance (*p* < 0.05). Figure S3. Morphology of spermatozoa from CT and RU roosters. (A) Representative pictures of spermatozoa from CT and RU roosters at Day 36 and 50. The blacks arrows show the different parts of spermatozoa morphology. Nucleus is highlighted with DAPI (white colour in the picture) and the tail with white light (200 spermatozoa/animal with *n* = 2CT and 2RU at Day 36 and *n* = 3CT and *n* = 3RU at Day 50). The white arrows show the abnormal head of the spermatozoa. * *p* < 0.05. (B) Quantification of the percentage of sperm with abnormal head morphology in spermatozoa from CT and RU roosters at Day 36 and Day 50 (200 spermatozoa/animal with *n* = 2CT and 2RU at Day 36 and *n*=3CT and *n*=3RU at Day 50). * *p* < 0.05. (C) Percentage of DNA damage staining on spermatozoa from CT and RU roosters (200 spermatozoa/animal with *n* = 2CT and 2RU at Day 36 and *n*=3CT and *n*=3RU at Day 50), * *p* < 0.05. Results are presented as means ± SEM. * *p* < 0.05. Figure S4. Steroidogenesis within the testis. Testes of RU and CT roosters were collected at Day 36 (end of RU exposure, *n* = 2CT and *n* = 2RU) and at Day 50 (14 days after RU exposure, *n* = 3CT and *n* = 3RU). (A) Testis testosterone quantification by ELISA assay (pg/mL). Stars (**) correspond to the unpaired *t*-test significance (*p* < 0.01). (B) Testis estradiol quantification by ELISA assay (pg/mL). Stars (*) corresponds to the unpaired *t*-test significance (*p* < 0.05). (C) Ratio between P450 SCC protein and vinculin protein amounts within the testis of CT and RU roosters. (D) Cholesterol level (mg/g of testis) in testes of RU and CT roosters collected at Day 36 and Day 50 (14 days after RU exposure). Stars (*) correspond to the unpaired *t*-test significance (*p* < 0.05).

## Abbreviations

AMPA	aminomethylphosphonic
ATP	Adenosine Triphosphate
3 β-HSD	3β-hydroxysteroid dehydrogenase
CASA	Computer-assisted sperm assessment
CT	Control
CYP17	cytochrome P450 17A1
CYP19	P450 aromatase
DBP	di(n-butyl) phthalate
EFSA	European Food Safety Authority
E2	estrogen
ER	estrogen receptor
eEF1α1	cycle-specific eukaryotic translation elongation factor 1 subunit alpha 1
EEM	early embryonic mortality
G	Glyphosate
GBHs	glyphosate-based-herbicides
IVOS	Integrated Visual Optical System
LEM	Late embryonic mortality
MRL	maximum residue level
NOAEL	No observable adverse effects level
P	progesterone
PBS	Phosphate Buffered Saline
P450scc	cytochrome P450 side-chain cleavage
ROS	Reactive Oxygen Species
RU	Roundup
StAR	steroidogenic acute regulatory
T	Testosterone
VAP	Average path velocity
VCL	curvilinear velocity
VSL	straight-line velocity
