# Supplementary material for: Chronic Dietary Exposure of Roosters to a Glyphosate-Based Herbicide Increases Seminal Plasma Glyphosate and AMPA Concentrations, Alters Sperm Parameters, and Induces Metabolic Disorders in the Progeny
Source: Toxics. 2021 Nov 24;9(12):318. doi: 10.3390/toxics9120318 (PMC8704617; doi:10.3390/toxics9120318)
Supplement: Supplementary file 1 [file toxics-09-00318-s001.zip › toxics-1454032-SM.pdf]

toxics

MDPI

Supplementary Materials: Chronic Dietary Exposure to a Glyphosate-Based Herbicide in Rooster Increases Seminal Plasma Glyphosate and AMPA Concentrations, Alters Sperm Parameters and Induces Metabolic Disorders in the Progeny

Loïse Serra, Anthony Estienne, Guillaume Bourdon, Christelle Ramé, Claire Chevaleyre, Philippe Didier, Marine Chahnamian, Souleiman El Balkhic, Pascal Froment and Joëlle Dupont

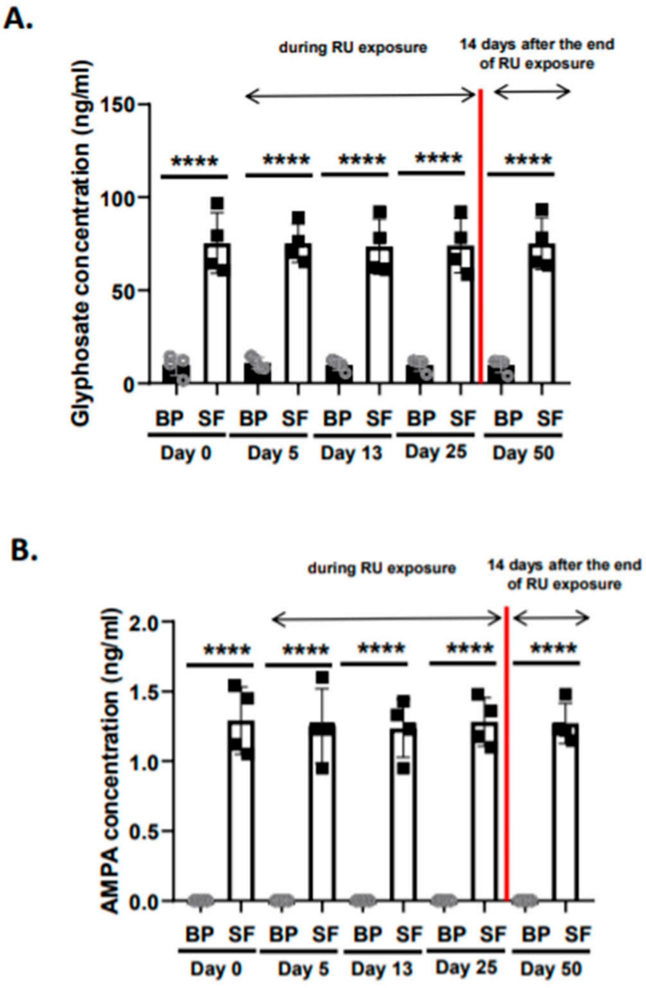

Figure S1. Morphology of testis and from CT and RU roosters. (**A**) Pictures of testis from CT and RU roosters at day 36 (at the end of RU exposure) and day 50 (14 days after RU exposure) with a light microscope, magnification x10 and x40 after staining with haematoxylin. (**B**) Measurement of the diameter (μm) of the seminiferous tubules of the testes of CT and RU roosters at day 36 (n = 4 testes from 2 CT and 2 RU animals) and Day 50 (n = 6 testes from 3 CT and 3 RU animals). Stars (****) correspond to the unpaired t-test significance ($p < 0.0001$)

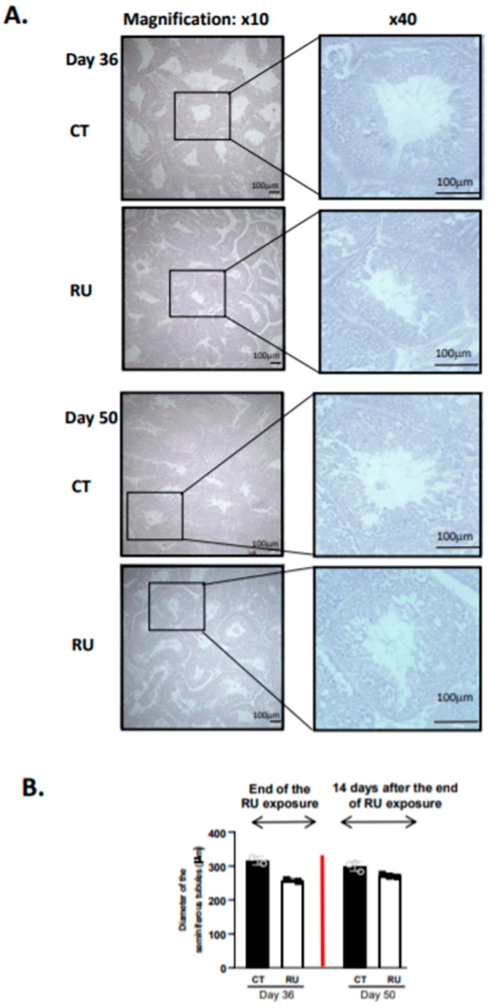

Figure S2. Morphology of spermatozoa from CT and RU roosters.**(A)** Representative pictures of spermatozoa from CT and RU roosters at day 36 and 50. The blacks arrows show the different parts of spermatozoa morphology. Nucleus is highlighted with DAPI (white colour in the picture) and the tail with white light (200 spermatozoa/animal with *n* = 2CT and 2RU at day 36 and *n* = 3CT and *n* = 3RU at day 50). The white arrows show the abnormal head of the spermatozoa. $p < 0.05$ (**B**) Quantification of the percentage of sperm with abnormal head morphology in spermatozoa from CT and RU roosters at day 36 and day 50 (200 spermatozoa/animal with *n* = 2CT and 2RU at day 36 and *n* = 3CT and *n* = 3RU at day 50). $p < 0.05$ **C**) Percentage of DNA damage staining on spermatozoa from CT and RU roosters (200 spermatozoa/animal with *n* = 2CT and 2RU at day 36 and *n* = 3CT and *n* = 3RU at day 50), $p < 0.05$. Results are presented as means ± SEM. *, $p < 0.05$

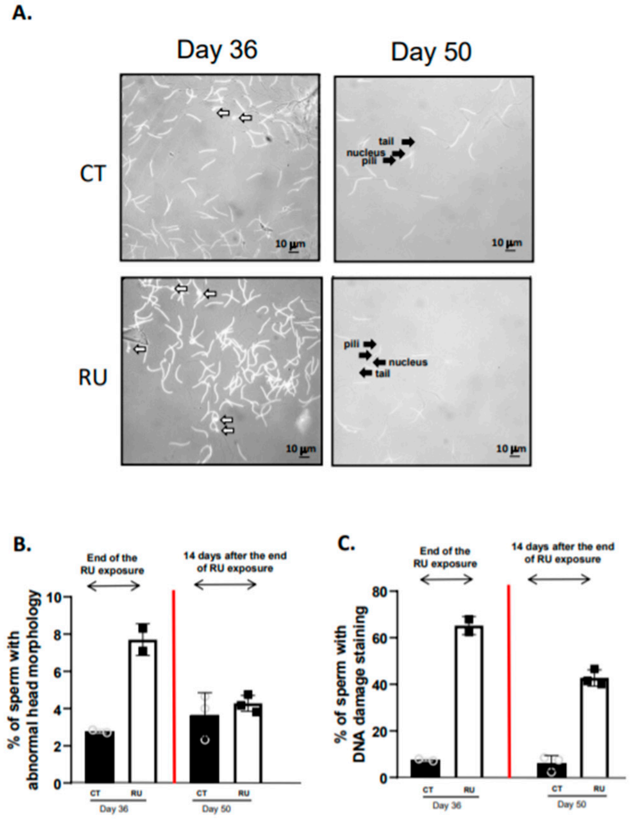

Figure S3. Morphology of spermatozoa from CT and RU roosters. (**A)** Representative pictures of spermatozoa from CT and RU roosters at day 36 and 50. The blacks arrows show the different parts of spermatozoa morphology. Nucleus is highlighted with DAPI (white colour in the picture) and the tail with white light (200 spermatozoa/animal with *n* = 2CT and 2RU at day 36 and *n* = 3 CT and *n* = 3RU at day 50). The white arrows show the abnormal head of the spermatozoa. (**B**) Quantification of the percentage of sperm with abnormal head morphology in spermatozoa from CT and RU roosters at day 36 and day 50 (200 spermatozoa/animal with *n* = 2 CT and 2 RU at day 36 and *n* = 3CT and *n* = 3RU at day 50). (**C**) Percentage of DNA damage staining on spermatozoa from CT and RU roosters (200 spermatozoa/animal with *n* = 2CT and 2RU at day 36 and *n* = 3CT and *n* = 3RU at day 50). Results are presented as means ± SEM.). White arrows show abnormal spermatozoa and black arrows indicate different structures of the spermatozoa (nucleus, tail and pili).

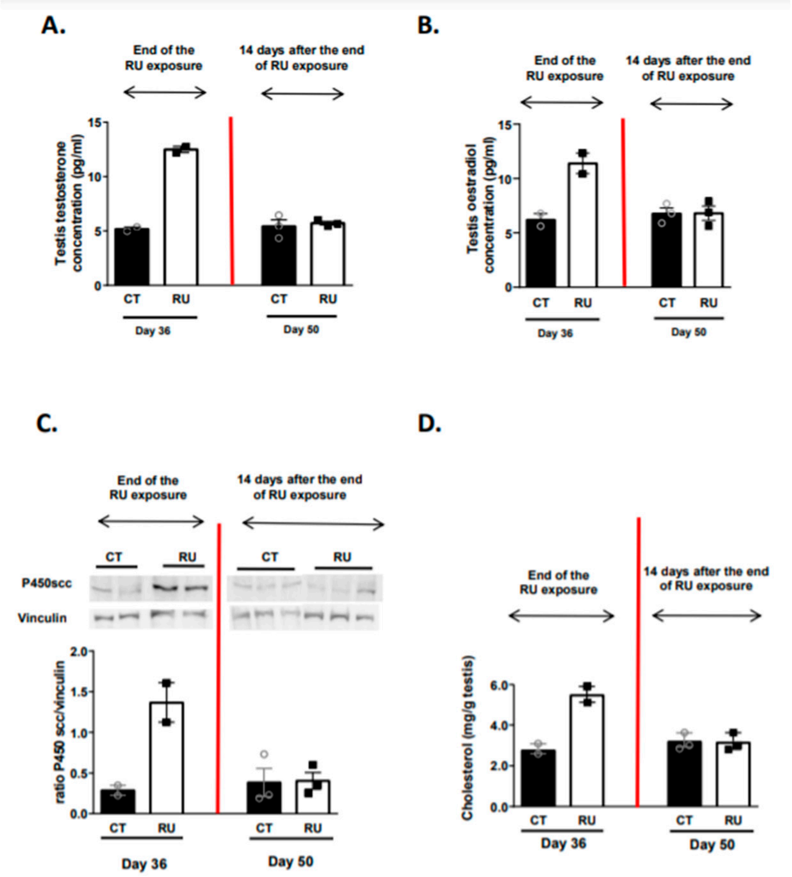

Figure S4. Steroidogenesis within the testis. Testes of RU and CT roosters were collected at Day 36 (end of RU exposure, *n* = 2CT and *n* = 2RU) and at Day 50 (14 days after RU exposure, *n* = 3CT and *n* = 3RU). (**A**) Testis testosterone quantification by ELISA assay (pg/mL). (**B**) Testis estradiol quantification by ELISA assay (pg/mL). (**C**) Ratio between P450 SCC protein and vinculin protein amounts within the testis of CT and RU roosters. (D) Cholesterol level (mg/g of testis) in testes of RU and CT roosters collected at Day 36 and Day 50 (14 days after RU exposure).
